# Vascular Auscultation of Carotid Artery: Towards Biometric Identification and Verification of Individuals

**DOI:** 10.3390/s21196656

**Published:** 2021-10-07

**Authors:** Rutuja Salvi, Patricio Fuentealba, Jasmin Henze, Pinar Bisgin, Thomas Sühn, Moritz Spiller, Anja Burmann, Axel Boese, Alfredo Illanes, Michael Friebe

**Affiliations:** 1 IDTM GmbH-Ingenieurgesellschaft für Diagnostischen und Therapeutische Medizintechnik mit Beschränkter Haftung, 45657 Recklinghausen, Germany; salvi@idtmt.de (R.S.); info@friebelab.org (M.F.); 2 Instituto de Electricidad y Electrónica, Facultad de Ciencias de la Ingeniería, Universidad Austral de Chile, Valdivia 5111187, Chile; 3 Fraunhofer Institute for Software and Systems Engineering, 44227 Dortmund, Germany; jasmin.henze@isst.fraunhofer.de (J.H.); pinar.bisgin@isst.fraunhofer.de (P.B.); anja.burmann@isst.fraunhofer.de (A.B.); 4 INKA-Innovation Laboratory for Image Guided Therapy, Otto-von-Guericke University, 39120 Magdeburg, Germany; thomas@surag-medical.de (T.S.); moritz@surag-medical.de (M.S.); Alfredo@surag-medical.de (A.I.); 5 SURAG Medical GmbH-Surgical Audio Guidance, 39120 Magdeburg, Germany; 6 MEDICS GmbH-Medical Innovation to Certification Services, 39114 Magdeburg, Germany; axel.boese@med.ovgu.de

**Keywords:** authentication, biometric, vascular sounds, carotid artery, spoofing

## Abstract

Background: Biometric sensing is a security method for protecting information and property. State-of-the-art biometric traits are behavioral and physiological in nature. However, they are vulnerable to tampering and forgery. Methods: The proposed approach uses blood flow sounds in the carotid artery as a source of biometric information. A handheld sensing device and an associated desktop application were built. Between 80 and 160 carotid recordings of 11 s in length were acquired from seven individuals each. Wavelet-based signal analysis was performed to assess the potential for biometric applications. Results: The acquired signals per individual proved to be consistent within one carotid sound recording and between multiple recordings spaced by several weeks. The averaged continuous wavelet transform spectra for all cardiac cycles of one recording showed specific spectral characteristics in the time-frequency domain, allowing for the discrimination of individuals, which could potentially serve as an individual fingerprint of the carotid sound. This is also supported by the quantitative analysis consisting of a small convolutional neural network, which was able to differentiate between different users with over 95% accuracy. Conclusion: The proposed approach and processing pipeline appeared promising for the discrimination of individuals. The biometrical recognition could clinically be used to obtain and highlight differences from a previously established personalized audio profile and subsequently could provide information on the source of the deviation as well as on its effects on the individual’s health. The limited number of individuals and recordings require a study in a larger population along with an investigation of the long-term spectral stability of carotid sounds to assess its potential as a biometric marker. Nevertheless, the approach opens the perspective for automatic feature extraction and classification.

## 1. Introduction

Authentication is the process of identifying and acknowledging a user’s identity to grant access to valuable information and property. It is an important aspect that needs to be considered for personal security, banking, travel, defense, and other principal sectors. Traditionally, authentication of personal data was done using personal ID cards or password-based encryption. However, these methods could easily be stolen or hacked into and used for malicious purposes [1,2]. More recently, biometric authentication was introduced as a security method to prevent such malpractice and allow for the identification of individuals [3,4,5].

Biometric techniques use the physiological or behavioral features of an individual to acknowledge, verify, and validate their identity [1,3]. Biometric sensing systems are vital tools in confirming these unique features for users. In order to qualify as a biometric authentication method, a parameter needs to be unique for every user, easily accessible at any given time, available lifelong, and easy to acquire [4]. Biometric systems function based on two different principles. The first is based on a one-to-many principle where the system matches the user input in an already existing database. The second is based on a one-to-one verification principle and matches a user input to a specific search parameter [3].

State-of-the-art biometric sensing methods include scans of fingerprints, voice, face, iris, gait, keystroke, signature, and handwriting [5]. These methods have advantages over traditional personal identification methods, for instance, considering they are distinctive from user to user and cannot be lost, stolen, or forgotten. However, there are still drawbacks and limitations of these methods and systems. As a unique trait, fingerprints suffer from drawbacks such as aging [6], skin disease [7], amputations [8], and spoofing with latex and modeling clay [9]. While an authentic voice recording could be used for unauthorized access, a voice sensing system is not efficient in cases where there is damage in vocal cords, infection in the larynx, or for a person with voice impairment, thus making the identification difficult. The facial recognition systems used as a biometric identification method have already addressed several issues such as variations in the face with changes in the skin, soft tissue, and skeleton of the face, as well as with the development of injuries and accidental face burns [10]. Additionally, technical challenges such as illumination and facial expression variation are tagged with face recognition [11]. This method has been previously attacked by fraudulent acts, such as obtaining access via a latex face mask [12]. The iris of a human also has a unique pattern and qualifies to be used as a biometric feature. Eventually, however, iris scans also underperform. There have been investigations regarding that the specifications of the iris change with time. Behavioral characteristics such as gait are certain to be affected depending on stormy weather conditions, capturing angle, alcohol consumption, leg injuries, and weight gain [13]. The signature and handwriting of a person can also be learned and forgotten. Additionally, the user needs to always sign in the same pattern, otherwise it would raise difficulties to acquire access to any system or resources. The main limitations and drawbacks concerning the above biometric traits are forgery, lifelong persistence, and effects of external environmental conditions. Further research in this field has offered a path to expand the knowledge in finding other biological characteristics that can overcome these limitations and can be used as biometric sensing tools. The new biological characteristic that proved to be possessing novel biometric information was the electrocardiogram (ECG), i.e., the ECG signal [14]. However, to acquire ECG signals, a complex setup with electrodes is required. The electrodes that are most commonly used are silver/silver chloride electrodes. However, the morphology of the signal is affected by change in the material of the electrode, by power line interference, and by baseline drift [15]. The heart sounds originating from the opening and closing of the heart valves also proved to have a biometric signature of the individual [16,17]. Heart sounds are complex audio signals that are difficult to tamper with or forge. These signals can be recorded with an electronic stethoscope [17]. The electronic stethoscope is a commonly used diagnostic tool used for the auscultation of, e.g., heart and lung sounds. For better visualization and study of these sounds by physicians, stethoscopes are designed with pre-processing circuitry and limited bandwidth. Therefore, the dynamic range of these electronic stethoscopes is very narrow, which lowers the capability of the device to identify an individual. This gives rise to the necessity to design and implement a new device that allows for a wide dynamic range to accommodate all information from the audio signals that is crucial in the identification of a person.

Therefore, we propose an audio acquisition device that allows for a wide dynamical range and provides a unique method of acquiring audio signals of the carotid artery that holds the potential to be used as a biometric sensing tool in the identification of individuals. The sound generated by the blood flow in the carotid arteries is used for diagnosis of carotid artery diseases. In this context, advanced signal processing and machine learning algorithms have been proposed to assess carotid artery stenosis [18,19,20,21]. However, to our knowledge, there are no studies that use carotid sounds as biometric information to classify or identify people. In this work, we show preliminary results of both qualitative and quantitative analyses which suggest that carotid sounds acquired with a simple device with a wide dynamic range could provide biometric information of a subject. To evaluate this approach, we acquired audio signals of the carotid artery of seven subjects with a recently proposed audio acquisition device at different intervals spaced over several weeks. For the qualitative analysis, wavelet-based signal processing methods were used that showed the stability of a number of patterns intra-subject and differences inter-subject. To analyze this further, we trained a simple convolutional neural network to distinguish between the different subjects based on these wavelet-based images. The results support the findings from the qualitative analysis.

## 2. Materials and Methods

Carotid sounds can satisfy the pre-requisites for being used as biometric information. They are universal, collectible with simple devices [22], and in healthy individuals involve a slow-changing curve over the lifetime [23], and our hypothesis is that they are distinctive. In other words, we postulate that the spectral information involved in carotid blood flow sounds extracted by a simple signal processing method, such as continuous wavelet transform (CWT) [24,25], allows for detecting the presence of biometric traits. Therefore, based on the carotid artery’s vascular auscultations, this innovative approach would allow for the biometric identification and verification of individuals with a simple handheld device or even with a smartphone microphone.

### 2.1. Audio Acquisition System

The main components of the acquisition system include the sensing hardware, a desktop application for real-time visualization of the acquired signals, a mechanical case to enclose the hardware, and a reliable skin-transducer interface to acquire high-quality signals. The device used for the audio acquisition was first introduced as an auscultation system for vascular sounds by Sühn et al. [26,27]. To summarize, the device consisted of two Knowles digital microphones (Knowles Electronics LLC, SPH0645LM4HB, Itasca, Illinois, USA) assembled on a custom-designed printed circuit board and with a raspberry pi (Raspberry Pi Foundation, Raspberry Pi Zero W, Cambridge, United Kingdom) as a host system. Both the host system and microphone were powered using a lithium potassium battery. As explained by Sühn et al., the two microphones were used to compare the differences in the signals when one was covered with an external membrane [27]. However, it was observed that there is no significant difference between the signals recorded by the two microphones. In this work, the same audio acquisition device was used. The custom-built printed circuit board with two microphones was replaced by the Adafruit I2S Knowles SPH0645LM4H-B microphone breakout board, containing only a single microphone. Due to this reason, the case of the device was also further developed and re-designed to fit the breakout board.

**Figure 1:**
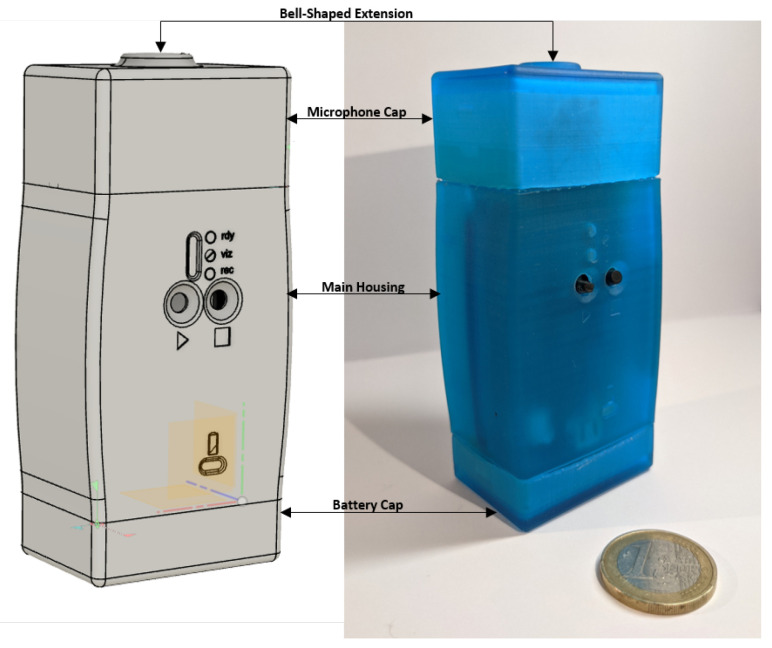
Mechanical 3D housing (**left**) and prototype (**right**).

Figure 1 shows the mechanical 3D model of the housing on the left and the 3D printed prototype on the right side of the image. It has three parts: the main case, the microphone cap, and the battery cap. The microphone cap has an extended bell-shaped design imitating the bell assembly of a stethoscope. Only the circumference of this bell assembly is in contact with the skin to acquire acoustic sounds from the surface of the neck. There is a cavity in the microphone cap through which the acoustic wave propagates to the microphone’s port. The microphone has an internal gain amplifier and a 18-bit sigma-delta analog-digital-converter, providing a digital output signal via I2S (inter-integrated circuit sound) codec. The acquired signals are stored in the .wav file format and transmitted to the desktop application by a self-generated Wifi network where they can be visualized and stored on a personal computer in real-time.

### 2.2. Data Acquisition

As explained above, the main idea is to study certain characteristics of the carotid sound signal that are associated with a subject and differ between subjects. For this operation, we used a real carotid sound database consisting of 890 recordings of 11 s in length, with a sampling frequency of $f_{s}=16$ kHz. These recordings were acquired from seven different users (U1, U2, …, and U7), who, to our knowledge, did not have vascular diseases. The collected data consist of recordings acquired from the left and right common carotid artery in equal proportion. In this work, the signals acquired from the left and right common carotid artery were analyzed independently.

Concerning the data collection, each user performed the acquisition process involving the following specifications.

- The carotid sound signals were recorded under controlled apnea. This allows for avoiding potential artifacts generated from the inspiration and expiration episodes involved in breathing episodes.
- The signals were acquired with a maximal range of seven months between the first and last recording, from December 2020 to June 2021. In this context, we assumed that the studied signal characteristics should not change in the short term for a subject with a normal health condition.
- Since the proposed device is in a prototyping stage, we decided to perform the signal acquisition by four devices (D1, D2, D3, and D4), which were designed and built as clones. The use of four clone devices allowed us to examine the signal characteristics from the four devices and thereby analyze the reliability of the extracted information of interest. The four devices were employed for all seven users, as presented in Table 1. This table shows the number of recordings acquired per user and per device.

### 2.3. Carotid Sound Signal Analysis

For the proposed approach, we extracted signal characteristics based on CWT spectral analysis [24,25]. On the one hand, we postulated that the carotid sounds involve complex characteristics in the spectral domain that contain significant information about the corresponding user. On the other hand, we considered that these characteristics can involve not only oscillatory dynamics but also a transient behavior for each cardiac cycle. In this perspective, considering these transient dynamics, CWT is better suited for the analysis compared with other traditional methods such as the Fourier transform [28]. Additionally, CWT allows for the analysis of the oscillatory characteristics of the signal. The signal processing techniques used in this work were implemented in the Matlab^®^ environment version 2021a.

**Figure 2:**
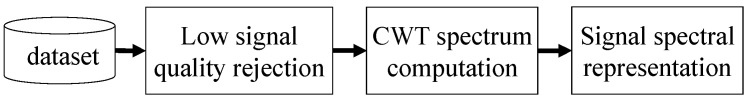
Proposed signal processing strategy.

Figure 2 presents our proposed processing strategy. As can be observed, once we had our dataset, we applied a low signal quality rejection to remove low-quality signals for the analysis. Then, the CWT spectrum was computed to obtain the spectral representation of the signal. These steps are explained next.

**Figure 3:**
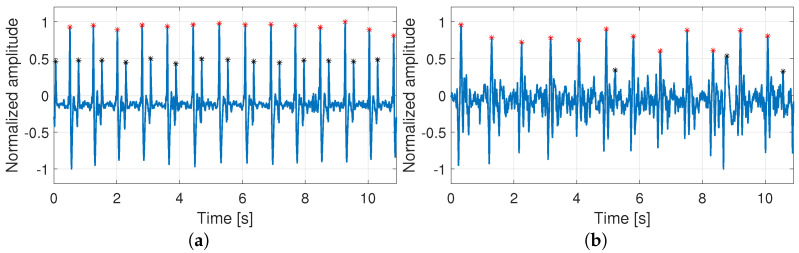
Examples of carotid sound recordings: (**a**,**b**) show recordings with high and low signal quality, respectively; identified peaks for S1 and S2 candidates are plotted in red and black, respectively.

After the signal acquisition, we applied a low signal quality rejection step in order to discard low-quality recordings from the analysis. This step involved a visual inspection of the carotid sound signal to recognize the presence of S1 and S2 events. These events correspond to the fundamental heart sounds originated at the beginning of the mechanical contraction (systole) and relaxation (diastole) of the heart ventricles [29]. In the case that recordings did not show marked S1 and S2 events, they were removed from the analysis. To better understand this criterion, Figure 3 shows two examples of carotid sound recordings. The x-axis corresponds to the time in seconds and the y-axis represents the signal amplitude normalized between −1 and 1. The signal presented in Figure 3a corresponds to a good quality recording, where S1 and S2 events can easily be recognized. In contrast, the example presented in Figure 3b represents a recording of low signal quality, where only some events of S2 can be correctly identified and most of the S2 events are not visually recognized. As a result, the first recording is selected and the second one is removed from the analysis. It is important to note that this manual quality assessment is performed in this work only to study the spectral characteristics of every user. Nevertheless, the biometric identification of the users does not depend on this preliminary assessment.

Based on this proposed criterion, from the 890 recordings, 881 were categorized as good quality recordings, which are presented in Table 2 per each user and each device. These selected recordings are considered for both qualitative and quantitative signal analyses, as will be explained in Section 3.

**Figure 4:**
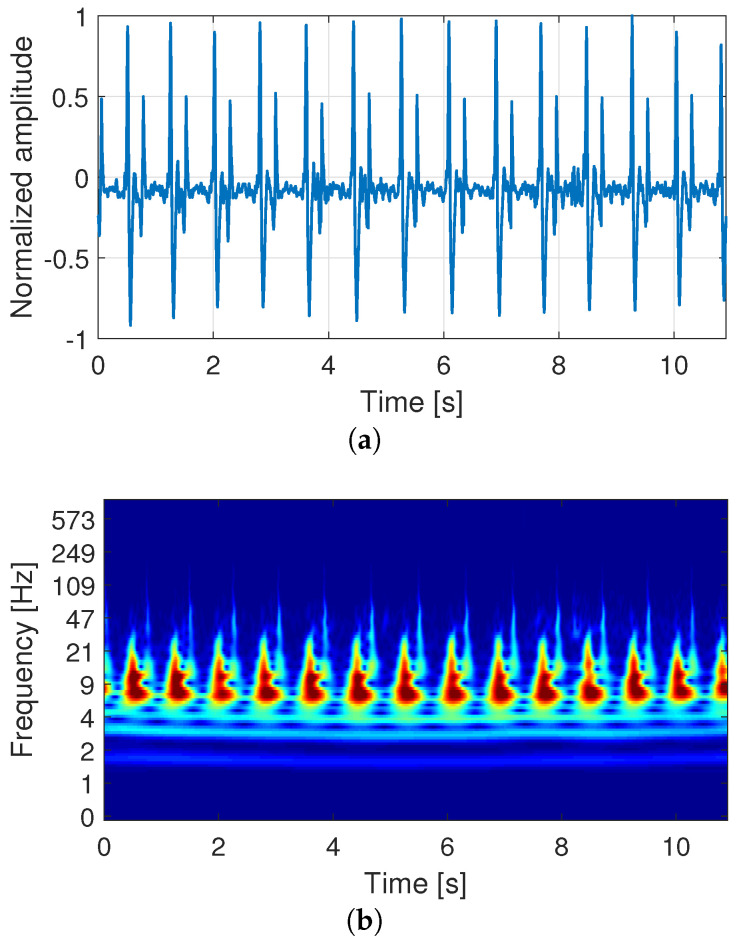
(**a**) Example of a raw signal of the carotid sounds recorded from user U1 during controlled apnea. (**b**) Continuous wavelet transform (CWT) spectrum computed from (**a**).

For the proposed signal analysis, we employed a time-scale spectral estimation based on CWT. It was applied using a Morse mother wavelet perfectly symmetric with a time-bandwidth product set to 60. Figure 4 shows an example of the employed signal processing method. Figure 4a depicts a raw carotid sound signal, where the y-axis represents the signal’s amplitude normalized between −1 and 1. Figure 4b shows the CWT spectrum computed from the signal of Figure 4a, where the y-axis represents pseudo-frequencies in Hz ordered in a logarithmic scale. It is important to note that the operation’s original frequency range was from 0 to $f_{s}/2=8000$ Hz, where $f_{s}$ corresponds to the sampling frequency of the recording. Nevertheless, the shown frequency range was reduced for better visualization of the frequency component of interest. In both figures, the x-axis corresponds to the time in seconds.

In Figure 4a, we can easily recognize the episodes S1 and S2 for each cardiac cycle, represented by the signal’s first and second prominent peaks, respectively. Figure 4b shows that the time-domain signal dynamics reflect different patterns in the spectral-domain, which involve a transient behavior for each cardiac cycle and are repeated over time. For each cardiac cycle, we can observe that S1 and S2 events involve the highest spectral energy, represented by the area in the red color. In contrast, the segment between S2 and S1 (diastole) involves lower spectral energy.

## 3. Results

Based on the methodology shown in Section 2, this section presents the results from the proposed analysis. First, Section 3.1 presents a qualitative analysis based on a visual examination of the spectral characteristics involved in the carotid sound signal. Then, in Section 3.2, we perform quantitative analysis to evaluate the discriminating capability of the spectral information based on the classification performance of the studied subjects.

### 3.1. Qualitative Spectral Analysis of the Carotid Sounds

**Figure 5:**
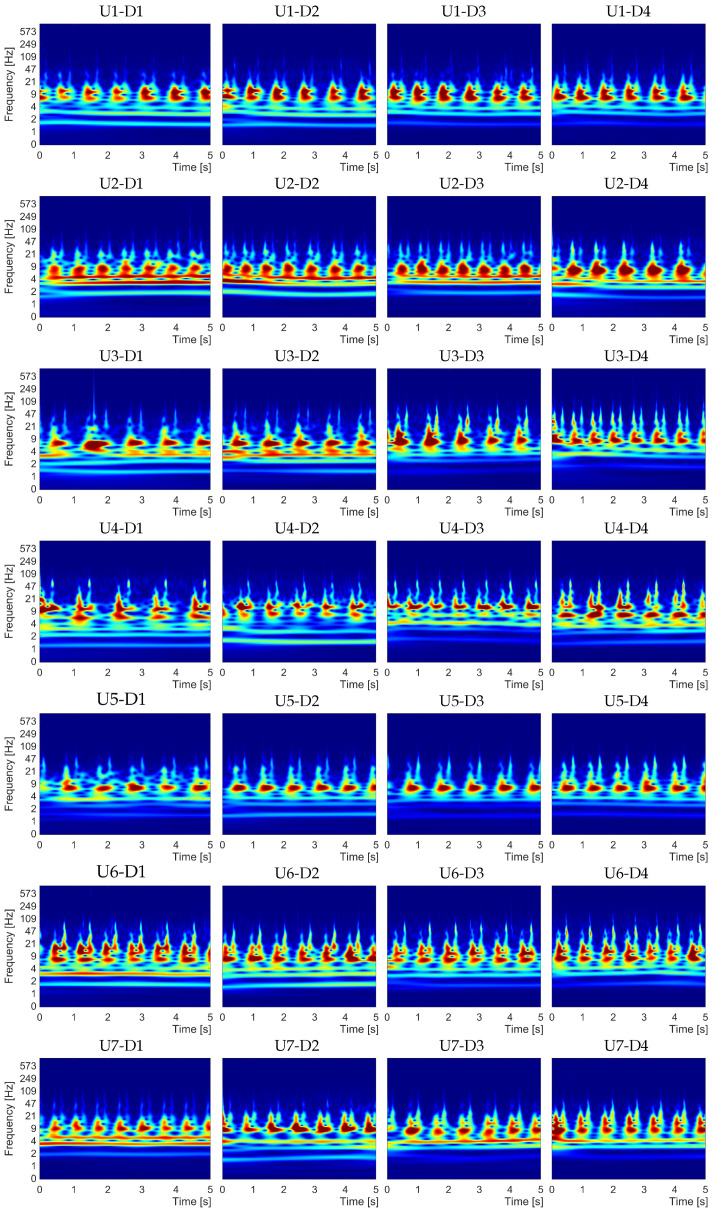
Examples of CWT spectral behavior of the carotid sounds for the seven users, organized as one user for each row. From the left to right, each column exhibits the CWT spectrum computed from recordings acquired by device D1, D2, D3, and D4.

Based on the spectral representation shown in Figure 4, as a preliminary step for the qualitative analysis, we examined the spectral characteristics of the studied carotid sound signals for each user. For this purpose, we considered the spectral representation of four recordings from each user, selected arbitrarily from each of the four employed devices. Figure 5 shows representative examples of carotid recordings selected from the left carotid artery of the seven users, specifically one user for each row. It is important to mention that this preliminary analysis considered recordings only from the left carotid artery. Nevertheless, the following spectral analysis applied to the carotid sound signal considers both carotid arteries.

In Figure 5, each example consists of CWT spectra computed from recordings acquired by the four devices, namely D1, D2, D3, and D4, and one device is presented for each column. It is important to note that the exhibited spectra involved only 5 s in length (from the 11 s) for a better visualization to compare their characteristics.

The main idea is to identify spectral dynamics for each cardiac cycle that prevail for the same user and differ between users. For this operation, we compared the spectral information of the representative examples presented in Figure 5. This display shows that the CWT spectra of the four devices involve particular characteristics that can be similar for the same user and can differ from one user to another. Specifically, for user U1, we observed that for most cardiac cycles, the maximal spectral energy (represented by red color) involved a region with a shape wider by around 7 Hz and narrower by 20 Hz. For user U2, the CWT spectrum involved a high energy frequency component focused between 4 and 9 Hz. For user U3, we observed that the spectral characteristics involved a frequency component between 4 and 20 Hz, whose main energy was located between 4 and 9 Hz. User U4 showed a more distributed spectral energy, segmented in two main areas, namely between 4 and 9 Hz, and between 9 and 21 Hz. User U5 presented more straightforward dynamics involving only one narrower main frequency component between 5 and 9 Hz. User U6, similar to U4, presented a spectrum segmented in two main frequency bands. For this user, each cardiac cycle exhibited episodes of high energy over 21 Hz and ended in two main tips around 100 Hz. Finally, for user U7, the spectrum involved a shape wider by around 9 Hz and narrower by 20 Hz.

In general, the signal dynamics reflected in the CWT spectrum showed similar characteristics for the same user. Although we can eventually find that there are slightly different dynamics for the same user, we can observe that the computed CWT spectrum can involve significant biometric information. As a next step, we proceeded to study these spectral characteristics to determine if they can help differentiate between subjects as biometric indicators.

In this context, we propose a global study of every cardiac cycle to examine their spectral characteristics for each user. First, we computed the average of the spectral energy for all cardiac cycles. Then, we analyzed the resulting spectra for the left and right carotid arteries. Considering that both carotid arteries can present significant physiological and anatomical differences from each other, e.g., artery wall thickness [30] and blood flow velocity [31], hypothetically, they might involve different sound dynamics for the same subject. Therefore, we decided to analyze the data recorded from the left and right carotid artery separately.

**Figure 6:**
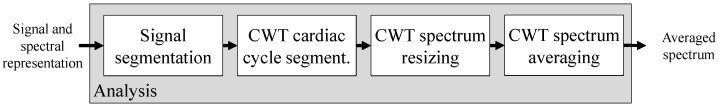
Proposed spectral analysis of the carotid sound signal.

For the proposed analysis, we postulated that the carotid sound signal’s specific spectral dynamics can be associated with a subject and can help discriminate between them. Figure 6 shows the main steps for the proposed operation, which are explained next.

**Figure 7:**
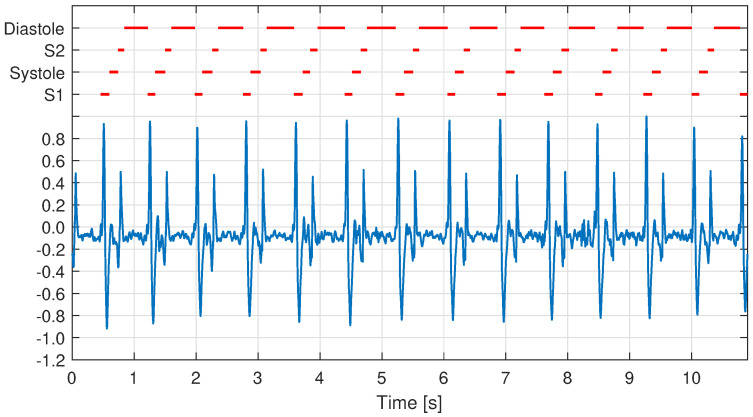
Example of the segmentation of S1, systole, S2, and diastole episodes of the carotid sounds applied to a signal acquired from user U1. The operation is based on the segmentation function for phonocardiogram recordings presented in [32].

- First, the carotid sound signal in the time-domain was segmented into the cardiac cycles. For this operation, we employed the segmentation function for phonocardiogram (PCG) recordings presented in [32]. This function assigns states to a PCG recording, specifically one state for each S1, systole, S2, and diastole episode, using a duration-dependent logistic regression-based Hidden Markov model. An example of this operation is presented in Figure 7, where the carotid signal and the assigned states are plotted in blue and red, respectively.
- Second, based on the signal segmentation performed in the time domain, we proceeded to segment the CWT spectrum for each cardiac cycle. It is essential to mention that for the spectral segmentation, we did not segment every episode (S1, systole, S2, and diastole) but rather we used these states to segment every cardiac cycle, starting with an episode of diastole and ending with an episode of S2. The number of cardiac cycles segmented from all the studied recordings for each user are presented in Table 3, which are shown separately for the left and right carotid artery.
- Third, to address the length difference between cardiac cycles, each segmented spectral matrix was re-sized in time with a length set to 1 s (16,000 samples, selected arbitrarily) and using the nearest-neighbor interpolation method. This operation allows for obtaining all the segmented spectra with the same size.
- Finally, all the segmented spectra were averaged pixel by pixel.

**Figure 8:**
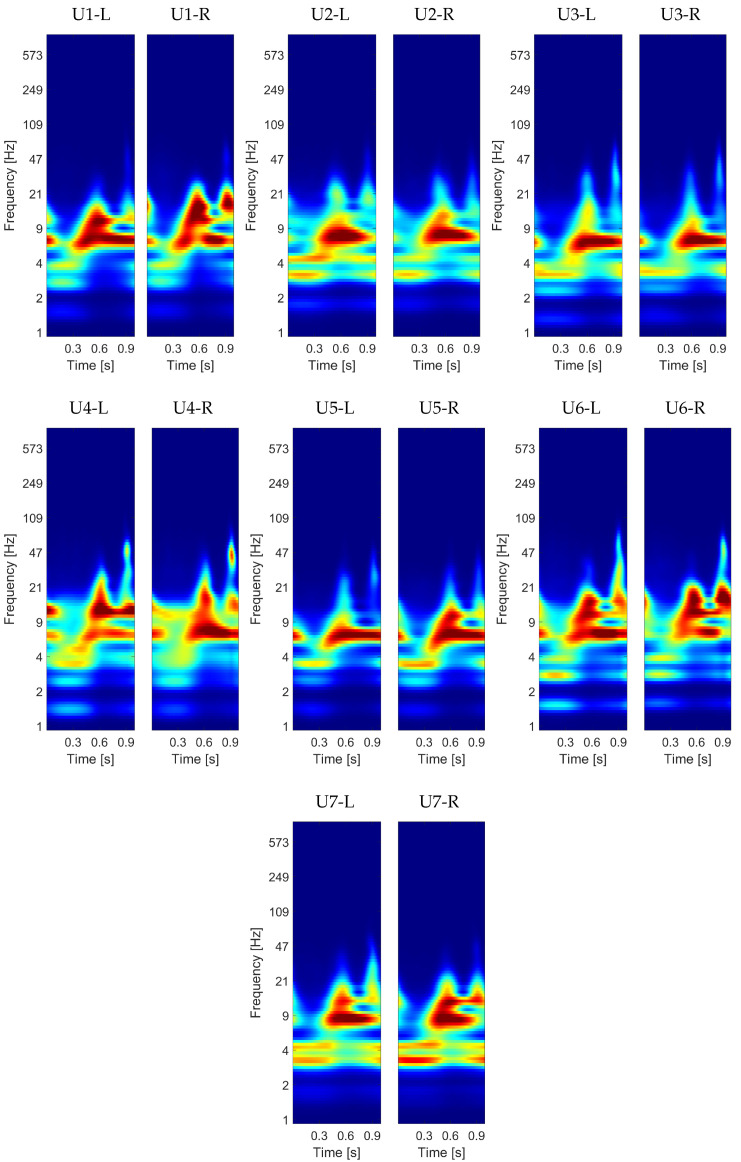
Averaged CWT spectrum considering all segmented cardiac cycles for the left (**L**) and right (**R**) carotid artery and for each user.

Figure 8 shows the global results from the proposed operation. Each pair of images corresponds to the resulting averaged spectrum computed from each user’s left and right carotid artery recording. This display allows us to compare the spectral behavior of the carotid sounds for the users based not only on a particular recording but also on all the studied cardiac cycles.

As can be observed in Figure 8, the spectra from the recordings acquired from the left and right carotid arteries show very similar spectral characteristics for each user. In general, the components of high spectral energy ranged, in terms of frequencies, between 1 and 100 Hz, and for the seven users, we observed a wide spectral component (from 0.3 to 1 s) around 7 Hz. Nevertheless, both spectra from the left and right carotid artery showed specific characteristics over 7 Hz that differed from one user to another.

In particular, for U1, the main spectral energy can be divided into two areas: one in the frequency range between 6 and 9 Hz, and the other between 9 and 21 Hz, the latter localized from 0.4 to 0.7 s. Unlike the previous case, for U2 and U3, this second frequency range showed a lower energy intensity, i.e., these users presented only the spectral component located around 7 Hz. For U3, however, this component presented a narrower band than for U2.

In contrast to the first three users, U4 showed a more distributed spectral energy, whose main component was located around 0.6 s and ended in an arrow-tip shape. Additionally, st around 0.9 s, we observed a high energy component in the range between 30 Hz and 100 Hz approximately, which was not present for the other users. Unlike the previous user, for frequencies higher than 9 Hz, the spectral energy of U5 considerably decreased. For U6, we recognized that the frequency band between 9 and 21 Hz involved two marked high energy episodes centered at 0.6 and 0.9 s. Finally, for U7, the frequency band around 7 Hz presented similar characteristics to the spectra of U2. Nevertheless, U7 showed higher spectral energy in the range of 9 and 21 Hz than U2.

All the characteristics described above are consistent with the preliminary analysis of the seven examples presented in Figure 5. In summary, the obtained results reveal that the proposed approach can be useful to discriminate between users based on only the spectral characteristics of their carotid sounds.

These qualitative results open perspectives for more objective analyses to probe them further. In this context, Section 3.2 presents a quantitative analysis in which we evaluate the discriminating capability of the studied data by a convolutional neural network based on CWT images, considering the left and right carotid artery separately.

### 3.2. Quantitative Analysis of the Carotid Sounds

Deep learning methods, particularly the use of convolutional neural network (CNN) models, are becoming increasingly popular in biometric recognition [33,34].

We developed a small CNN based on the segmented cardiac cycles to learn the relatively simple decision boundary provided by the CWT representation. As a basis, we obtained data as listed in Table 3. Before feeding the data into the convolutional layers of the network, the data was preprocessed. Pixel values were often unsigned integers in the range from 0 to 255. Although these pixel values can be presented directly to the neural network models in their raw format, this can lead to problems during modeling, e.g., slower training of the model. To prevent this, the pixel values were scaled to the range from 0 to 1. The proposed small CNN consists of 3 convolutional layers followed by 2 fully connected layers, whereas the last fully connected layer corresponds to the seven subjects/classes (see Table 4). A small CNN was designed to learn less complex decision boundaries in the transferred domain to achieve better generalization capability and simultaneously to avoid overfitting.

A stratified 5-fold cross-validation with 10 repetitions was used to obtain a reliable estimation of the network’s performance. Therefore, samples were randomly distributed among the five folds at each replicate, ensuring that the ratio between the different subjects/classes remained the same at each fold to maintain the same ratio of training to testing for each iteration. Here, 5 folds were chosen to obtain a larger test data set of 20% to derive a quantitative result. Ten repetitions were chosen to obtain fifty different results, the average of which is the final accuracy of the classification task. A learning rate of 0.001 was considered during the 10 training epochs alongside a batch size of 32. We tried higher values for the training epochs but no improvements were observed.

From the classification results, the true-positive (TP), true-negative (TN), false-positive (FP), and false-negative (FN) values were calculated for each subject/class. TP is the number of samples from one subject/class that were correctly classified and TN is the number of test samples that were correctly classified as not belonging to that subject/class. FP is the number of test samples that were incorrectly classified as belonging to that subject/class, while FN is the number of test samples that were incorrectly classified as not belonging to that subject/class [35,36]. The confusion matrix of a classifier summarizes the performance measures TP, TN, FP, and FN of our model. The confusion matrix can be further used to extract other performance measures per class, such as sensitivity (SEN), specificity (SPE), precision (PRE), and F1-score (F1). Sensitivity measures the proportion of TP that is correctly identified as such. Specificity measures the proportion of TN that is correctly identified as such. Precision indicates the proportion of correctly predicted positives relative to the total of all the results predicted as positive. The F1-score is the harmonic mean of precision and sensitivity, and is often used as a summary metric [35,37]. On the x-axis are the predicted labels and on the y-axis are the true labels of the samples in our test set. Ideally, a perfect classifier would result in a confusion matrix where values different from 0 are only on the diagonal, i.e., in a case where a model correctly classifies all test samples for all the seven classes present.

**Figure 9:**
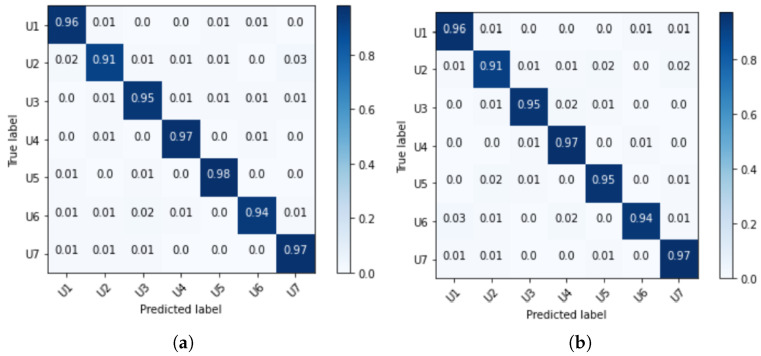
Mean normalized confusion matrix (**a**) for the left and (**b**) right carotid artery.

Figure 9 shows the mean normalized confusion matrix: (a) for the samples from the left and (b) for the samples from the right carotid artery. When examining both carotids, it is clear that both sides enable a detection of all users with a probability of >91%. In addition to the confusion matrix, the evaluation metrics were calculated and are in the following table.

From Table 5, it can be seen that the sensitivities for the different subjects did not differ significantly between the experiments based on samples from the left and right sides. One exception can be seen for user 5; the algorithm detected user 5 with a sensitivity of (98.08±1.64)% from the left data set. However, for the right carotid, it could correctly match (95.35±3.19)% of the images. When examining the other users, we noticed that the biometric recognition based on the left or right carotid artery was equally good. We achieved a minimum classification probability of (90.99±4.07)% (user 2-L). In addition, it was notable that a specificity of more than 98.98% was detected for all users with a very low standard deviation of <±0.67%. The PRE values differed minimally among the different users with respect to the left or right carotid artery. This was due to TP, i.e., those correctly identified as corresponding users. The difference for user 5 can also be seen here. With the left carotid, an accuracy of (97.54±1.73)% could be achieved. Since F1 can be calculated from SEN and PRE, we can see some similarity in the values here, as well. We obtained the highest F1 for the left carotid artery of user 5: (97.79±0.98)%. We obtained the worst F1 for user 2 for both the left and right carotid arteries. From these results, we can see that it is possible to distinguish between users using the CWT images. This is even possible when only one carotid artery is used.

In addition to these metrics, as listed in the table, the overall accuracy (ACC) was also calculated. Accuracy can be calculated using the formula [36]:

(1) $$ACC=\frac{TP+TN}{TP+TN+FP+FN}$$

This calculates to (95.86±0.83)% for the experiments based on samples from the left carotid artery and (95.69±0.90)% based on samples from the right carotid artery. By and large, the trained model can identify the seven subjects regardless of the side.

## 4. Discussion

This work focused on the analysis of carotid sound signals based on the hypothesis that the spectral information of the carotid blood flow sounds involve significant information useful to discriminate between individuals based on the presence of biometric traits. For this purpose, we performed a qualitative signal analysis based on a time-scale CWT spectral estimation to examine the spectral characteristics of the studied signals.

The obtained results show that it is possible to detect certain signal characteristics in the spectral domain that can distinguish one user from another. In the quantitative analysis, we were able to identify the individual users using CNN. Here, we also considered both the left and right carotid artery. Results revealed that it is even possible to distinguish between the users by any side of the carotid artery. However, the parameters of the blood flow in the carotid artery would change with age, would change if the person develops a stenosis, or if the person undergoes a surgery. In this case, the person needs to recollect the signals again to reset the baseline. Additionally, in this work, the carotid acoustic signal was only analyzed based on seven users and a limited number of recordings. Therefore, additional studies are needed to further prove our proposed hypothesis. As a next step, we propose to consider a higher number of both recordings and users, which could provide more representative and reliable results. This approach opens perspectives for automatic feature extraction and classification of subjects based on the spectral characteristics of their carotid sound recordings. Furthermore, the studied biometrical information could be used clinically to obtain and highlight differences from a previously established personalized audio profile and subsequently to provide information on the source of the deviation as well as its effects on the individual’s health status.

## Figures and Tables

**Table 1:** Number of recordings acquired per each user and each device.

User	D1	D2	D3	D4
U1	20	20	94	20
U2	20	20	24	20
U3	46	22	22	22
U4	40	40	40	40
U5	20	80	20	20
U6	20	20	20	20
U7	40	40	40	40

**Table 2:** Number of recordings of good quality selected for the analysis per each user and each device.

User	D1	D2	D3	D4
U1	20	20	94	20
U2	20	20	24	20
U3	46	46	22	22
U4	39	40	37	39
U5	20	80	20	20
U6	20	20	20	20
U7	40	38	39	39

**Table 3:** Number of segmented cardiac cycles for the left (L) and right (R) carotid artery and for each user.

User	U1	U2	U3	U4	U5	U6	U7
Left	907	572	583	824	770	491	1102
Right	921	574	604	860	742	503	1084

**Table 4:** Proposed small CNN architecture.

Layer Number	Type	Output Shape	Parameter
1	Image input	134×134×134	0
2	Convolution	132×132×132	896
3	Max-pooling	66×66×32	0
4	Convolution	64×64×32	9248
5	Max-pooling	32×32×32	0
6	Convolution	30×30×32	9248
7	Max-pooling	15×15×32	0
8	Fully connected	128	921,728
9	Fully connected	7	903

**Table 5:** Evaluation metrics for the left (L) and right (R) carotid artery and for each user.

	SEN	SPE	PRE	F1
U1-L	(96.38±2.32)%	(99.20±0.56)%	(96.28±2.44)%	(96.29±1.41)%
U1-R	(96.46±2.79)%	(99.31±0.56)%	(96.82±2.37)%	(96.56±1.48)%
U2-L	(90.99±4.07)%	(99.03±0.46)%	(92.17±3.30)%	(91.46±1.76)%
U2-R	(91.46±4.38)%	(98.98±0.67)%	(91.93±4.56)%	(91.53±2.37)%
U3-L	(94.54±2.54)%	(99.34±0.43)%	(94.86±3.10)%	(94.64±1.60)%
U3-R	(95.50±2.97)%	(99.52±0.49)%	(96.44±3.34)%	(95.89±1.89)%
U4-L	(97.05±1.64)%	(99.42±0.47)%	(96.95±2.28)%	(96.97±1.19)%
U4-R	(97.40±2.19)%	(99.26±0.62)%	(96.35±2.79)%	(96.82±1.31)%
U5-L	(98.08±1.64)%	(99.57±0.32)%	(97.54±1.73)%	(97.79±0.98)%
U5-R	(95.35±3.19)%	(99.31±0.52)%	(95.88±2.89)%	(95.54±1.48)%
U6-L	(98.98±0.38)%	(99.47±0.45)%	(95.13±3.75)%	(94.60±2.01)%
U6-R	(93.98±3.79)%	(99.37±0.41)%	(94.21±3.30)%	(94.00±2.08)%
U7-L	(96.90±2.19)%	(99.12±0.58)%	(96.76±1.99)%	(96.80±1.20)%
U7-R	(97.09±1.71)%	(99.22±0.56)%	(97.05±1.93)%	(97.04±0.97)%

## Data Availability

The data was only used for this study and is not publicly available.
